# Material Basis and Mechanism of Chansu Injection for COVID-19 Treatment Based on Network Pharmacology and Molecular Docking Technology

**DOI:** 10.1155/2021/7697785

**Published:** 2021-10-11

**Authors:** Yong Xu, Wenpan Peng, Di Han, Zhichao Wang, Fanchao Feng, Xianmei Zhou, Qi Wu

**Affiliations:** ^1^Affiliated Hospital of Nanjing University of Chinese Medicine, Nanjing 210029, China; ^2^Department of Respiratory and Critical Medicine, Jiangsu Province Hospital of Chinese Medicine, Nanjing 210029, China; ^3^Department of Physiology, Xuzhou Medical University, Xuzhou 221009, China

## Abstract

**Purpose:**

The clinical efficacy of Chansu injection for COVID-19 treatment has been confirmed. Its mechanism of action remains unclear. We used network pharmacology and molecular docking technology to explore the potential material basis and mechanism of action of Chansu injection for COVID-19.

**Methods:**

The main components of Chansu injection were determined using HPLC. The PharmMapper, SwissTargetPrediction, SEA, and TCMID databases were used to screen for the active ingredients and therapeutic targets of Chansu injection, while the OMIM and GeneCards Suite databases were used to search for COVID-19-related targets. The STRING database was used for protein-protein interaction (PPI) network construction and topological analysis, while DAVID was used for Gene Ontology (GO) and Kyoto Encyclopedia of Genes and Genomes (KEGG) pathway enrichment analyses of the core targets. The main active compounds of Chansu injection were docked with 3CL protease, ACE2, RdRp, and spike protein.

**Results:**

The three Chansu injection compounds were identified using HPLC. A total of 236 drug-related targets and 16,611 disease-related targets were identified, and 77 common targets were determined through mapping. The PPI mapping results revealed that 16 core targets were obtained through topological analysis and screening. Furthermore, GO and KEGG pathway enrichment analyses revealed that the PI3K and JAK-STAT signaling pathways are the major pathways. The molecular docking results suggest that the three Chansu injection components have high binding energies to the S protein.

**Conclusions:**

The potential mechanism of Chansu injection for COVID-19 involves multiple targets and pathways, thereby providing a scientific basis for its clinical application and further research.

## 1. Introduction

Coronavirus disease 2019 (COVID-19) caused by severe acute respiratory syndrome coronavirus 2 (SARS-CoV-2) emerged in China in November 2019 [1]. This disease has become a global pandemic with severe health issues around the world [2, 3]. This is the third pandemic that has occurred during the last 20 years of the 21st century, the first of which was the SARS pandemic in 2003, and the second, Middle East respiratory syndrome (MERS) pandemic in 2015 [4–6]. Worldometers real-time statistics show that as of February 1, 2021, SARS-CoV-2 has caused more than 100 million infections and more than 2.22 million deaths. As a result, it is considered a public health emergency of international concern [7–9].

SARS-CoV-2 is the seventh coronavirus found to infect humans. Similar to SARS-CoV and MERS-CoV, SARS-CoV-2 belongs to the *β*-subtype coronavirus [10]. Its genetic similarity to that of the bat coronavirus is 96%, and it shares the same cell receptor, angiotensin-converting enzyme II (ACE2), found in SARS-CoV. SARS-CoV-2 is a single-stranded RNA with the largest RNA virus [11, 12]. It can trigger an antiviral immune response upon entry into organisms, causing slight or moderate respiratory symptoms that are difficult to notice in most patients. Severe cases display excessive inflammatory responses, leading to severe progressive pneumonia and even acute respiratory distress syndrome, septic shock, coagulation disorders, and multiorgan failure [13, 14]. Owing to its severity and rapid progression, there is an urgent need to develop novel medicines for the emerging coronavirus [15].

Although inactivated viral vaccines, adenoviral vector vaccines, recombinant protein vaccines, and mRNA vaccines have been intensively developed in various countries, they are also in use and have achieved good efficiency indicators [16]. However, vaccination and development are also difficult [17]. The problem with common viral vector vaccines is that many people have already developed an immune response to these viral vectors, and these neutralizing antibodies against adenovirus are likely to have an impact on the effectiveness of the vaccine. Inactivated viral vaccines not only require adjuvants but also often require multiple doses to boost immunity. The efficacy of the ground-breaking mRNA vaccine in humans needs to be further tested, and whether it can be delivered through the mucous membranes of the respiratory tract is also a question worth exploring. In the face of long vaccine development and vaccination cycles, and with little success in acute COVID-19 patients, there is an urgent need for an effective antiviral drug that can mitigate disease progression in acute patients or in vaccine-free areas.

However, to date, no medicines specific for coronavirus treatment are available. Before the COVID-19 pandemic in China, the coverage and effectiveness of Chinese medicines were greater than 90% [18]. Results of clinical observation revealed that Chinese medicine could effectively mitigate disease progression, stop the progression from mild to severe, increase the recovery rate, decrease the death rate, and promote rehabilitation [19]. The full participation of Chinese medicine has become a highlight of the anti-COVID-19 campaign in China and has contributed to the pandemic's global campaign. As a result, Chinese medicines have been increasingly acknowledged.

Chansu is obtained from the excreta of the skin and postauricular glands of *Bufo bufo gargarizans*. Chansu injection is a prescription Chinese medicine, its main component is a derivative of indole base, and this product is a colorless to light yellow clear liquid, often used for clearing heat and detoxifying effect. It fights inflammation and infection and regulates immunity [20, 21]. Chansu injection is now being widely administered to inhibit bacteria, fight viruses, suppress pain, reduce inflammation, inhibit the proliferation of tumor cells, increase the patient's albumin level after radiochemical therapy, and induce tumor cell apoptosis. Favorable clinical therapeutic efficiency and safety assessments have also been acquired in the ancillary treatment of cancer and chronic hepatitis. The main active ingredients in Chansu, such as bufalin, resibufogenin, and cinobufagin, have very strong antiviral effects [22, 23]. In a clinical trial, Fen et al. found that Chansu injection significantly improved the respiratory function of patients with severe COVID-19, suggesting a marked clinical effect and no side effects [24].

**Figure 1:**
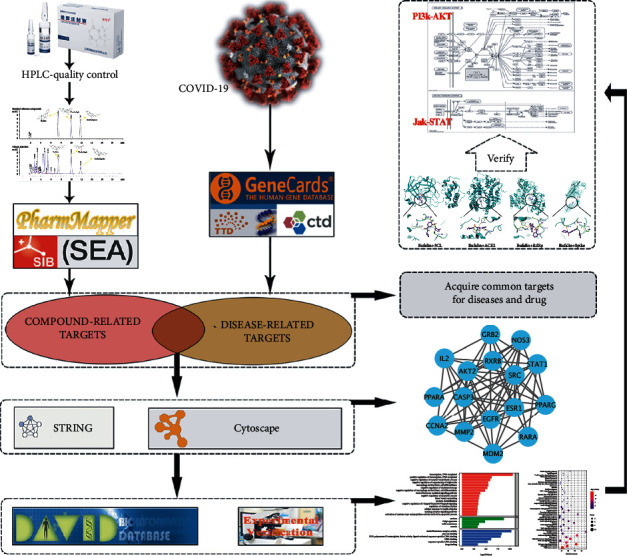
Flowchart of the study.

Network pharmacology is an emerging discipline that integrates the technology and content of polypharmacology, computational biology, and network analysis [25]. Because the integrality and systematicity of network pharmacology are consistent with the Chinese medicine theory [26], it has been widely used in the prediction and characterization of potential targets and active ingredients of Chinese medicine [27]. Molecular docking technology is a theoretical simulation approach to predict the binding pattern and affinity of medicines using computers to study the receptors' characteristics as well as the interaction between receptors and medicinal molecules [28]. In this study, we attempted to identify the effective ingredients and targets of Chansu injection for COVID-19 treatment using a network pharmacological method. Molecular docking was also applied in investigating the binding capacity of protein receptors to small medicine molecules based on geometric mapping and energy mapping, a key-and-lock principle. The material basis and molecular mechanism of Chansu injection for COVID-19 treatment were explored. A flowchart of the research methodology is presented in Figure 1.

## 2. Materials and Methods

### 2.1. Database

PharmMapper (https://lilab-ecust.cn/pharmmapper/), Universal Protein (UniProt, https://www.uniprot.org/) database, similarity ensemble approach (SEA, https://sea.bkslab.org) database, Swiss database (SwissTargetPrediction, https://www.sib.swiss), STRING (https://string-db.org), the Database for Annotation, Visualization, and Integrated Discovery (DAVID, https://david.ncifcrf.gov), GeneCards database platform (https://www.genecards.org), Comparative Toxicogenomics Database (CTD, https://ctd.mdibl.org), and Therapeutic Target Database (TTD, https://bidd.nus.edu.sg/group/ttd/ttd.asp) were used.

### 2.2. Ingredient Analysis

The Chansu injection sample was supplied by the Pujin Pharmaceutical Cooperation (Jiangsu, China). The chromatographic conditions and system compatibility tests included the Agilent Zorbax SB-C18 chromatographic column (column length 250 mm, inner diameter 4.6 mm, and particle size 5 *μ*m) and the mobile phase containing acetonitrile and 0.1% ammonium acetate solution (50 : 50). The detection wavelength was 296 nm. Theoretical plate counts were calculated according to the bufalin level peak, which was not less than 3,000. To prepare the control solution, an appropriate amount of bufalin, resibufogenin, and cinobufagin was calibrated precisely and 1.6 *μ*g each of bufalin, resibufogenin, and cinobufagin were added to 1 mL of 50% acetonitrile aqueous solutions. The control solution (20–50 *μ*L) and 50 *μ*L of the test samples were loaded into high-performance liquid chromatography (HPLC) equipment, and the calculations were derived with an external method. Each 1 mL of sample should contain 1.2–4.8 *μ*g of bufalin (C_24_H_34_O_4_), cinobufagin (C_26_H_34_O_6_), and resibufogenin (C_24_H_32_O_4_).

### 2.3. Acquisition of Potential Targets

Potential targets of the active ingredients of Chansu injection were searched online on PharmMapper, SEA, and SwissTargetPrediction, using the index words bufalin, resibufogenin, and cinobufagin. The combined results were considered potential targets.

### 2.4. Acquisition of Targets for COVID-19

The index words coronavirus disease, coronavirus infections, and severe acute respiratory syndrome were searched in CTD, TTD, and GeneCards Suite, respectively. The index word search was conducted on January 20, 2021. The combined results of the three databases were used, and duplications were removed to obtain the corresponding targets for COVID-19.

### 2.5. Acquisition of the Crosstalk Targets of the Disease and Medicine

The corresponding targets of Chansu injection related to COVID-19 were mapped using the R language data package (version 3.6.1). The crosstalk targets were considered potential targets for COVID-19 treatment with Chansu injection.

### 2.6. Network Construction

A medicine-target-disease network diagram was prepared by introducing the acquired information of crosstalk targets and active ingredients into the Cytoscape software (v3.7.2). Thereafter, a topological analysis was performed.

### 2.7. Acquisition of the PPI Diagram

The acquired crosstalk targets were recorded in the STRING database using the genus *Homo sapiens*. The free targets were deleted, and the resultant file, string_interactions.tsv, was introduced into the Cytoscape software for topological analysis. Core targets were determined according to the degree and centrality of the intermediate value.

### 2.8. GO and KEGG Enrichment Analysis

The function of the core targets was described and annotated to investigate its pathway. Kyoto Encyclopedia of Genes and Genomes (KEGG) and Gene Ontology (GO) enrichment analyses were conducted using the DAVID database. For GO enrichment analysis, molecular function (MF), biological process (BP), and cellular component (CC) were used to confine and describe genes. The potential signaling pathway for COVID-19 treatment using Chansu injection was determined through KEGG enrichment analysis.

### 2.9. Molecular Docking

The molecular structure of the active ingredients of Chansu injection was downloaded from PubChem, while those of 3CL protease, ACE2, RdRp, and spike protein were downloaded from the PDB database. The data were processed using MGLTools 1.5.6 and saved as a pdbqt file. The active sites and grid box coordinates of 3CL protease, ACE2, RdRp, and spike protein were defined according to their built-in ligands. The size of each box was 30 × 30 × 30 grids. Small-molecule proteins were docked using AutoDock Vina 1.1.2; molecular structures were stereotyped using PyMOL; and the top scoring conformations were mapped and analyzed using Maestro 11.9.

We used ChemBioDraw Ultra 17.0 to draw the structures of compounds, which were then converted to 3D structures using ChemBio3D Ultra 17.0 and optimized using MMFF94 force fields. The 3D structures of RdRp, 3CL, ACE2, and spike were downloaded from the RCSB Protein Data Bank (https://www.rcsb.org), and we selected the 3D structures of RdRp (PDB ID: 6ld3), 3CL (PDB ID: 6LU7), ACE2 (PDB ID: 1R42), and spike (PDB ID: 6acc) were used as the proteins for docking in this project. Both proteins and compounds were converted to PDBQT format using AutoDockTools 1.5.6 [29, 30]. AutoDock Vina 1.1.2 [31] was used for the molecular docking study. To increase the accuracy of the calculations, we set the parameter exhaustiveness to 20. The default values were used for all parameters except where noted. Finally, the conformation with the highest scoring value was selected for analysis of the results using Free Maestro 11.9.

## 3. Results

### 3.1. Active Ingredients of Chansu Injection

**Figure 2:**
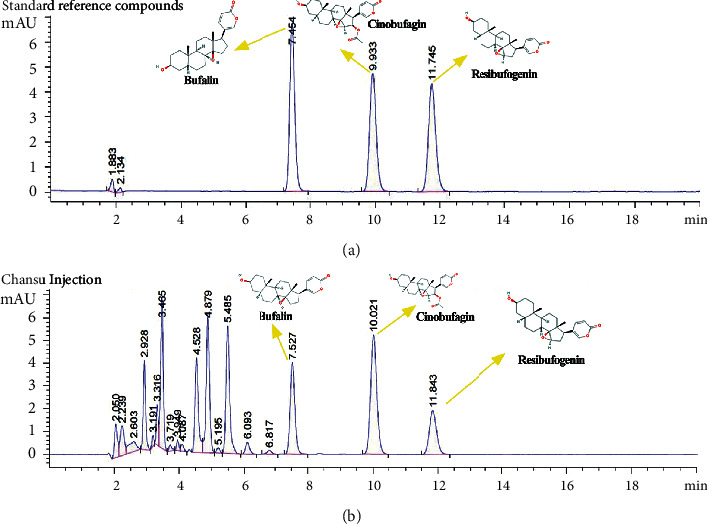
HPLC chromatograms of the components of Chansu injection.

The major components of Chansu injection were analyzed using HPLC. On comparison with standard reference compounds, three compounds were identified: bufalin, resibufogenin, and cinobufagin. Representative chromatograms of the standard reference compounds (Figure 2(a)) and Chansu injection components (Figure 2(b)) are shown in Figure 2.

### 3.2. Potential Targets of Chansu Injection

The active ingredients of bufalin, resibufogenin, and cinobufagin were searched online in PharmMapper, SEA, and Swiss databases, and the results were combined and duplicates were eliminated to obtain a total of 236 potential targets.

### 3.3. Crosstalk Targets of Chansu Injection and COVID-19

**Figure 3:**
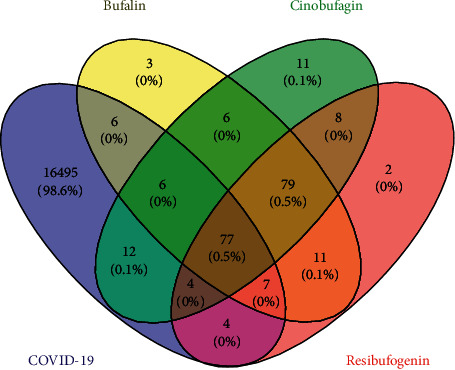
Venn diagram.

By searching GeneCards, CTD, and TTD, a total of 16,611 disease-related targets were obtained (7,927 from GeneCards, 14,774 from CTD, and 2 from TTD). When the potential targets of Chansu injection were mapped to disease-related targets, a total of 77 crosstalk targets were identified. In particular, COVID-19 was found to have 96, 99, and 92 crosstalk targets with bufalin, cinobufagin, and resibufogenin, respectively (Figure 3).

### 3.4. Medicine-Target-Disease Network Diagram

**Figure 4:**
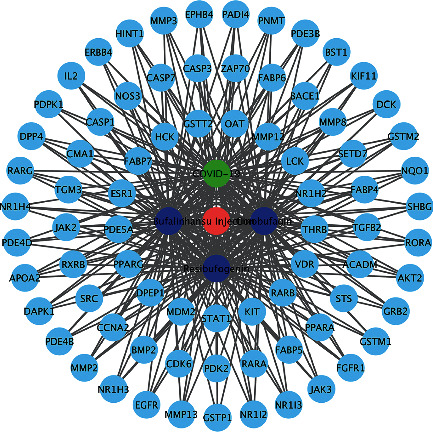
Network diagram of the drug-target-disease.

In the medicine-target-disease network diagram (Figure 4), the outer light blue nodes represent targets, the inner dark blue nodes represent the active ingredients of Chansu injection, the red node represents medicines, and the green node represents diseases. Topological analysis of the network diagram showed that there were 82 nodes and 137 connecting lines. Furthermore, the network centralization, heterogeneity, and density were 0.199, 1.096, and 0.032, respectively. This suggests an important link between Chansu injection and COVID-19 and motivated us to proceed to the next step in our analysis.

### 3.5. PPI Diagram and Core Target Filtration

**Figure 5:**
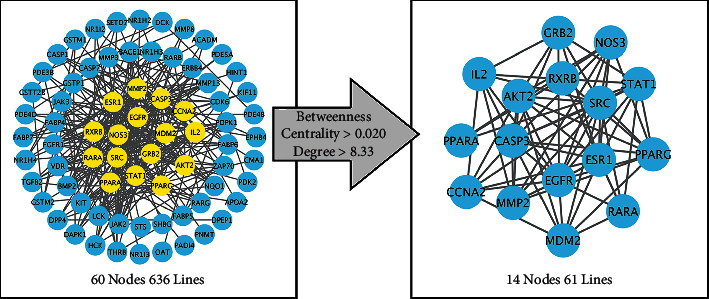
PPI diagram and filtration of the core targets.

Information concerning the crosstalk targets of the disease and drug was introduced into the STRING database for analysis using the genus *Homo sapiens*. Four free targets were deleted, and the resultant file “string_interactions.tsv” was introduced into the Cytoscape software (v3.7.2) to draw the protein-protein interaction (PPI) diagram. Thereafter, a topological analysis was performed. The nodes with retention value and the centrality of intermediate values above the mean values are the core targets (Figure 5).

As shown in Table 1, core targets were obtained, namely, AKT2, GRB2, EGFR, CCNA2, RXRB, RARA, STAT1, PPARG, ESR1, SRC, NOS3, IL2, PPARA, MMP2, MDM2, and CASP3.

### 3.6. GO and KEGG Enrichment Analysis

**Figure 6:**
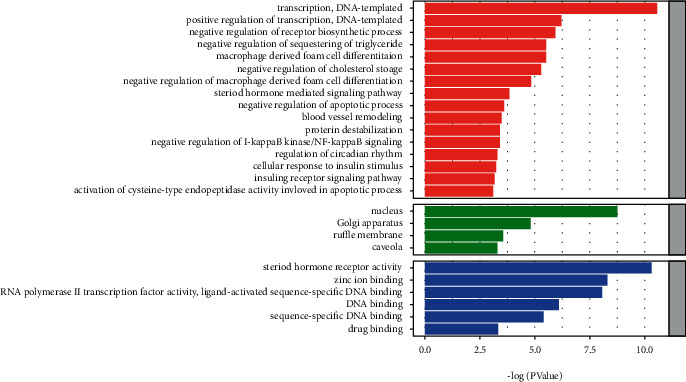
Results of GO enrichment analysis.

**Figure 7:**
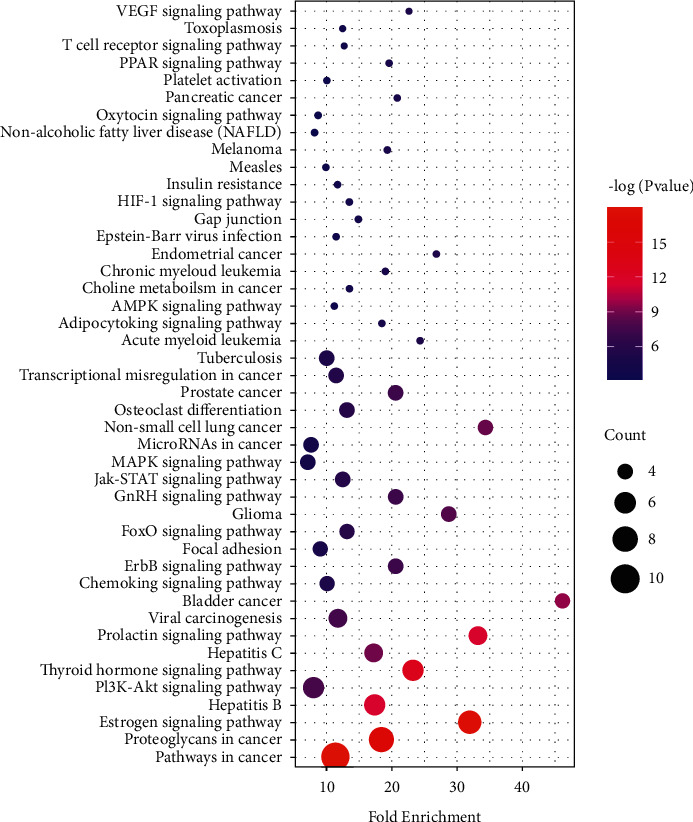
Results of KEGG enrichment analysis.

Based on the GO enrichment analysis, a total of 26 entries had a *P* value less than 0.05, including 16 BP, 4 CC, and 6 MF (Figure 6). These entries are involved in cell proliferation, modulation of apoptosis and programmed death, cascading response of intracellular signals, immune responses, nucleic acid metabolism processes, transcription, and translation. Based on the KEGG enrichment analysis, the phosphoinositide 3-kinase (PI3K-Akt) and JAK-STAT signaling pathways differed significantly and were closely related to viral infection (Figure 7).

### 3.7. Molecular Docking

The 3CL sequence is highly conserved in coronaviruses and is essential for the normal function of SARS-CoV-2 [32]. The high affinity of ACE2 for the extracellular domain structure of the novel coronavirus S protein is 15 nM (equilibrium dissociation constant), which is 10–20 times higher than the affinity of ACE2 for the extracellular domain structure of the SARS coronavirus S protein [33]. The high affinity of ACE2 may help the virus to spread easily from human to human, and it is therefore an important target.

**Figure 8:**
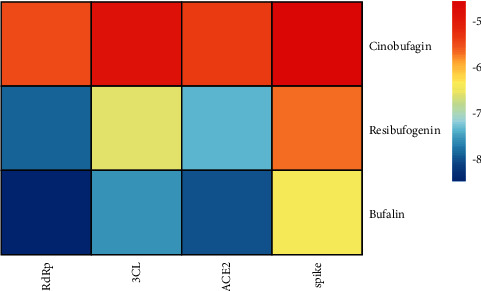
Diagram of the molecular docking efficiency.

**Figure 9:**
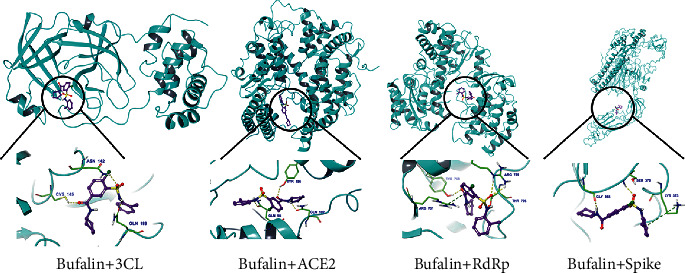
Molecular docking of Chansu injection to 3CL protease, ACE2, RdRp, and spike protein.

As shown in Figure 8, bufalin had the highest binding efficiency to 3CL protease, ACE2, RNA-dependent RNA polymerase (RdRp), and spike protein, with an absolute binding efficiency above 7, indirectly suggesting that the Chansu injection has a good therapeutic effect on SARS-CoV-2. The molecular docking diagram of bufalin and virus proteins is shown in Figure 9.

## 4. Discussion

COVID-19 is a global pandemic in the 21^st^ century that has greatly threatened human health and safety [34]. Chinese medicines can enhance the self-immunity and reconstruction capacity of the human body when used to prevent infectious diseases, thereby maintaining homeostasis [35]. They function to balance immunity, eliminate inflammation, and avoid or delay inflammation outburst by regulating immunity, inflammation, endocrine function, signal transduction, and other BPs and inhibiting the production of bacterial endotoxins through multiple ingredients and targets [36]. Chinese medicines have a unique advantage and provide effective control strategies in severe conditions, such as global pandemics, which lack effective treatments.

Previously, bufalin, the main active ingredient of Chansu, was found to have a broad-spectrum antiviral activity that can inhibit mouse hepatitis virus, feline infectious peritonitis virus, MERS-CoV, and vesicular stomatitis virus infecting host cells via the ATP1A1-mediated Src signaling pathway [37, 38]. In addition, ATP1A1 plays a crucial role in the infection of host cells by Ebola and respiratory syncytial virus (RSV) [39, 40].

A 50-sample clinical [24] study showed that Chansu injection could significantly improve the respiratory function of patients with severe COVID-19. In fact, the PaO_2_/FiO_2_ and ROX of 95.2% patients in the treatment group were improved, while those in the control group improved by 68.4% and 73.7%, respectively. The respiration-supported recovery time of the treatment group was one day shorter than that of the control group. Meanwhile, the results of the safety index analysis showed that Chansu injection did not have evident toxic side effects at the tested dosage.

As Chansu injection can effectively treat COVID-19, network pharmacology was used to study the molecular mechanism of Chansu injection, as with other comprehensive pharmacological analyses of medicinal plants [25], which might be involved in the regulation of cell proliferation, cell apoptosis, programmed cell death, intracellular signaling cascade response, immune response, nucleic acid metabolism, transcription, translation, and other BPs in COVID-19 treatment. Based on the KEGG enrichment analysis results, the PI3K-Akt and JAK-STAT signaling pathways may play a critical role. Furthermore, the molecular docking results suggested that bufalin, one of the active ingredients in Chansu injection, has a very high binding efficiency to 3CL protease, ACE2, and RdRp, and spike protein, with an absolute binding efficiency above 7, suggesting that the active ingredients in Chansu injection may have stopped viral entry into host cells and blocked its binding to ACE2 and the binding of the SARS-CoV-2 protein to the hydrolase of SARS-CoV-2 3CL protease, thereby terminating viral RNA duplication. This study has laid the foundation for future confirmation of the molecular mechanisms of Chansu injection in COVID-19 treatment.

At present, the role of the PI3K-Akt signaling pathway in various diseases has been confirmed. In humans, the PI3K-Akt pathway regulates multiple cellular and biological processes, such as tumor immunity, virus infection, and inflammatory recovery. PI3K is an intracellular phosphatidylinositol kinase (PIK) that also has Ser/Thr kinase and PIK activities, which participate in the regulation of various cellular functions, such as proliferation, differentiation, and glucose transportation [41, 42]. The PI3K-Akt signaling pathway was found to be involved in the infection processes of different types of viruses, including RNA and DNA viruses, which mainly regulate viral activity in the early infection stage, recognize host cell infection, and mediate cell transformation [43–46].

Iranian scholars found that viruses depend on two types of receptors to enter cells: ACE2 and CD147 [47]. Other molecular factors on the cell surface that facilitate viral genome entry into host cells include TMPRSS2 and furin protease. CD147 and furin protease both help induce the PI3K/Akt signal transduction pathway. Furthermore, the endocytosis of SARS-CoV-2 is mediated by clathrin, and this pathway is regulated by PI3K/Akt signal transduction. Once the virus binds to ACE2, the cell membrane sinks and viral endocytosis occurs. A reduction of ACE2 on the cell surface helps increase the level of serum angiotensin II (Ang II), which has been proven in COVID-19 patients. However, the binding of Ang II to its type I receptor (AT1R) activates this signaling pathway, thereby regulating the SARS-CoV-2 infection to host cells and virus duplication in infected cells. Therefore, the PI3K/Akt signaling pathway could regulate the activation of activator protein-1(AP-1) and nucleic factor-*κ*B (NF-*κ*B), reduce the expression of inflammatory cytokines such as interleukin-6 (IL-6) and tumor necrosis factor alpha (TNF-*α*), inhibit the cascading reaction of an inflammatory outburst, and promote the absorption of inflammatory cells and tissue recovery.

The JAK/STAT signaling pathway plays a crucial role in the regulation of cell growth, survival, and pathogen resistance [48–51]. Professor Yaoxing Wu et al. [52] found that type I interferon pretreatment could significantly interfere with the duplication of SARS-CoV-2, suggesting a certain sensitivity of SARS-CoV-2 to type I interferon cells. The induced interferon was released to regulate the JAK-STAT-mediated expression of multiple downstream antiviral cytokines (such as IFN-*γ* and IL-3) and inhibit virus duplication and amplification. The M^pro^ in SARS-CoV-2 could promote autophagic degradation of the transcriptomic factor STAT1 to weaken the entrance of STAT1 into the nucleus. This process inhibits the activity of type I interferon cells, reduces the expression of the downstream active ingredient of the JAK-STAT pathway, and helps COVID-19 patients acquire a natural immune escape.

A study [53] by June's research group at the University of Pennsylvania (USA) found that the cytokine release syndrome (CRS) is the primary lethal cause for SARS and MERS patients, as well as COVID-19 patients. When a coronavirus enters the body, monocytes, macrophages, and dendritic cells secrete IL-6 and other inflammatory factors. The downstream activity of IL-16 mainly includes the classical cis and trans pathways. In the cis pathway, IL-6 forms a complex with the MIL-6 membrane and gp130 and activates the JAK and STAT3 pathways. MIL-6 is mainly expressed in immune cells; therefore, this pathway can activate immune cells to trigger CRS. In the trans pathway, the highly concentrated IL-6 binds to the soluble IL-6R and forms a dipolymer with gp130. As gp130 is widely expressed on the surface of all cells, the downstream JAK-STAT3 pathway can activate cells that do not express MIL-6R, such as epithelial cells. Furthermore, they may increase levels of vascular endothelial growth factor (VEGF), monocyte chemotactic protein-1 (MCP-1), and interleukin-8 (IL-8) levels, as well as release of IL-6 and reduction of E-cadherin, eventually leading to acute respiratory distress syndrome (ARDS).

## 5. Conclusion

In summary, in this study, we determined the binding efficiency of Chansu injection to the main protein of the novel coronavirus and explored the functioning mechanism of this Chinese medicine for COVID-19 treatment using network pharmacology and molecular docking based on highly effective clinical studies. The results showed that Chansu injection could regulate virus duplication, activate immune cells, and alleviate inflammatory responses through the PI3K-Akt and JAK-STAT signaling pathways. The Chansu injection treats patients with COVID-19 by acting on SARS-CoV-2 and ACE2. The results of this study provide theoretical and experimental evidence for COVID-19 treatment using Chansu injection.

## Figures and Tables

**Table 1:** Core targets of Chansu injection.

Gene name	Protein name	UniProt ID
AKT2	RAC-beta serine/threonine-protein kinase	P31751
GRB2	Growth factor receptor-bound protein 2	P62993
EGFR	Epidermal growth factor receptor	P00533
CCNA2	Cyclin-A2	P20248
RXRB	Retinoic acid receptor RXR-beta	P28702
RARA	Retinoic acid receptor alpha	P10276
STAT1	Signal transducer and activator of transcription 1-alpha/beta	P42224
PPARG	Peroxisome proliferator-activated receptor gamma	P37231
ESR1	Estrogen receptor	P03372
SRC	Proto-oncogene tyrosine-protein kinase Src	P12931
NOS3	Nitric oxide synthase	P29474
IL2	Interleukin-2	P60568
PPARA	Peroxisome proliferator-activated receptor alpha	Q07869
MMP2	72 kDa type IV collagenase	P08253
MDM2	E3 ubiquitin-protein ligase Mdm2	Q00987
CASP3	Caspase-3	P42574

## Data Availability

The data and materials used to support the findings of this study are available from the corresponding author upon request.

## Abbreviations

COVID-19: Coronavirus disease 2019
TCMSP: Traditional Chinese Medicine Systems Pharmacology Database and Analysis Platform
DL: Drug-like
OB: Oral bioavailability
PPI: Protein-protein interaction
GO: Gene Ontology
KEGG: Kyoto Encyclopedia of Genes and Genomes
BP: Biological process
CC: Cellular component
MF: Molecular function.

## Supplementary Materials

Supplementary Materials
*Supplementary Table 1.* Detailed data of association and attribution of genes, ingredients, medicine, and diseases in Figure 4.
