# Supplementary material for: Material Basis and Mechanism of Chansu Injection for COVID-19 Treatment Based on Network Pharmacology and Molecular Docking Technology
Source: Evid Based Complement Alternat Med. 2021 Oct 11;2021:7697785. doi: 10.1155/2021/7697785 (PMC8523246; doi:10.1155/2021/7697785)
Supplement: Supplementary Materials — Supplementary Table 1. Detailed data of association and attribution of genes, ingredients, medicine, and diseases in Figure 4. [file 7697785.f1.docx]

**Supplementary Material**

**Supplementary Table 1.** Detailed data of association and attribution of genes, ingredients, medicine and diseases in Figure 4.

| **Node1** | **Node2** | **Net** | **Chansu Injection** | **Bufalin** | **mol** | **Chansu Injection** | **Cinobufagin** | **mol** | **Chansu Injection** | **Resibufogenin** | **mol** |
| --- | --- | --- | --- | --- | --- | --- | --- | --- | --- | --- | --- |
| COVID-19 | IL2 | disease | Bufalin | STS | target | Cinobufagin | APOA2 | target | Resibufogenin | BMP2 | target |
| COVID-19 | DPP4 | disease | Bufalin | BMP2 | target | Cinobufagin | CASP7 | target | Resibufogenin | STS | target |
| COVID-19 | CASP3 | disease | Bufalin | CASP3 | target | Cinobufagin | BMP2 | target | Resibufogenin | CASP7 | target |
| COVID-19 | NR1H4 | disease | Bufalin | ESR1 | target | Cinobufagin | STS | target | Resibufogenin | APOA2 | target |
| COVID-19 | ESR1 | disease | Bufalin | APOA2 | target | Cinobufagin | MMP3 | target | Resibufogenin | CASP3 | target |
| COVID-19 | CASP7 | disease | Bufalin | KIF11 | target | Cinobufagin | GSTP1 | target | Resibufogenin | BACE1 | target |
| COVID-19 | NOS3 | disease | Bufalin | MMP3 | target | Cinobufagin | ESR1 | target | Resibufogenin | KIF11 | target |
| COVID-19 | CASP1 | disease | Bufalin | CASP7 | target | Cinobufagin | CCNA2 | target | Resibufogenin | PPARG | target |
| COVID-19 | MMP2 | disease | Bufalin | THRB | target | Cinobufagin | RORA | target | Resibufogenin | ESR1 | target |
| COVID-19 | NR1H3 | disease | Bufalin | PPARG | target | Cinobufagin | EPHB4 | target | Resibufogenin | THRB | target |
| COVID-19 | JAK2 | disease | Bufalin | BACE1 | target | Cinobufagin | CASP3 | target | Resibufogenin | EGFR | target |
| COVID-19 | PPARG | disease | Bufalin | SHBG | target | Cinobufagin | SRC | target | Resibufogenin | GSTP1 | target |
| COVID-19 | SRC | disease | Bufalin | RORA | target | Cinobufagin | BACE1 | target | Resibufogenin | NR1I3 | target |
| COVID-19 | EGFR | disease | Bufalin | NQO1 | target | Cinobufagin | SHBG | target | Resibufogenin | NQO1 | target |
| COVID-19 | CCNA2 | disease | Bufalin | EPHB4 | target | Cinobufagin | MMP13 | target | Resibufogenin | PDPK1 | target |
| COVID-19 | BMP2 | disease | Bufalin | NOS3 | target | Cinobufagin | THRB | target | Resibufogenin | PDK2 | target |
| COVID-19 | MDM2 | disease | Bufalin | SRC | target | Cinobufagin | EGFR | target | Resibufogenin | MMP13 | target |
| COVID-19 | CDK6 | disease | Bufalin | MMP13 | target | Cinobufagin | PDPK1 | target | Resibufogenin | SRC | target |
| COVID-19 | MMP13 | disease | Bufalin | PDE5A | target | Cinobufagin | PPARG | target | Resibufogenin | SHBG | target |
| COVID-19 | GSTP1 | disease | Bufalin | PDE4B | target | Cinobufagin | KIF11 | target | Resibufogenin | PDE4B | target |
| COVID-19 | NR1I2 | disease | Bufalin | EGFR | target | Cinobufagin | NQO1 | target | Resibufogenin | RORA | target |
| COVID-19 | NR1I3 | disease | Bufalin | DPP4 | target | Cinobufagin | FABP6 | target | Resibufogenin | NOS3 | target |
| COVID-19 | JAK3 | disease | Bufalin | PDPK1 | target | Cinobufagin | NOS3 | target | Resibufogenin | PDE4D | target |
| COVID-19 | STAT1 | disease | Bufalin | CCNA2 | target | Cinobufagin | NR1H2 | target | Resibufogenin | CCNA2 | target |
| COVID-19 | PDK2 | disease | Bufalin | FGFR1 | target | Cinobufagin | CMA1 | target | Resibufogenin | EPHB4 | target |
| COVID-19 | FGFR1 | disease | Bufalin | MMP8 | target | Cinobufagin | LCK | target | Resibufogenin | PNMT | target |
| COVID-19 | GSTM1 | disease | Bufalin | GSTP1 | target | Cinobufagin | RARG | target | Resibufogenin | RARG | target |
| COVID-19 | GRB2 | disease | Bufalin | MMP12 | target | Cinobufagin | PNMT | target | Resibufogenin | JAK3 | target |
| COVID-19 | AKT2 | disease | Bufalin | PDE4D | target | Cinobufagin | PDE5A | target | Resibufogenin | NR1H3 | target |
| COVID-19 | RARA | disease | Bufalin | PDE3B | target | Cinobufagin | NR1I3 | target | Resibufogenin | FABP7 | target |
| COVID-19 | RORA | disease | Bufalin | DCK | target | Cinobufagin | PDE4D | target | Resibufogenin | LCK | target |
| COVID-19 | FABP5 | disease | Bufalin | CDK6 | target | Cinobufagin | JAK3 | target | Resibufogenin | DPP4 | target |
| COVID-19 | PPARA | disease | Bufalin | FABP7 | target | Cinobufagin | PDE4B | target | Resibufogenin | FABP6 | target |
| COVID-19 | KIT | disease | Bufalin | JAK3 | target | Cinobufagin | PDK2 | target | Resibufogenin | NR1H2 | target |
| COVID-19 | RARB | disease | Bufalin | FABP5 | target | Cinobufagin | CDK6 | target | Resibufogenin | NR1H4 | target |
| COVID-19 | STS | disease | Bufalin | FABP6 | target | Cinobufagin | MDM2 | target | Resibufogenin | PPARA | target |
| COVID-19 | ACADM | disease | Bufalin | NR1H4 | target | Cinobufagin | PPARA | target | Resibufogenin | ERBB4 | target |
| COVID-19 | VDR | disease | Bufalin | NR1H3 | target | Cinobufagin | FABP7 | target | Resibufogenin | MDM2 | target |
| COVID-19 | TGFB2 | disease | Bufalin | PNMT | target | Cinobufagin | NR1H3 | target | Resibufogenin | MMP8 | target |
| COVID-19 | SHBG | disease | Bufalin | NR1I3 | target | Cinobufagin | DPP4 | target | Resibufogenin | CDK6 | target |
| COVID-19 | THRB | disease | Bufalin | PDK2 | target | Cinobufagin | PDE3B | target | Resibufogenin | RARB | target |
| COVID-19 | NQO1 | disease | Bufalin | LCK | target | Cinobufagin | FGFR1 | target | Resibufogenin | PDE3B | target |
| COVID-19 | NR1H2 | disease | Bufalin | PPARA | target | Cinobufagin | NR1H4 | target | Resibufogenin | HCK | target |
| COVID-19 | FABP4 | disease | Bufalin | MDM2 | target | Cinobufagin | MMP8 | target | Resibufogenin | ZAP70 | target |
| COVID-19 | LCK | disease | Bufalin | FABP4 | target | Cinobufagin | ERBB4 | target | Resibufogenin | FABP5 | target |
| COVID-19 | GSTM2 | disease | Bufalin | RARG | target | Cinobufagin | PADI4 | target | Resibufogenin | ACADM | target |
| COVID-19 | MMP12 | disease | Bufalin | NR1H2 | target | Cinobufagin | JAK2 | target | Resibufogenin | DPEP1 | target |
| COVID-19 | DCK | disease | Bufalin | DPEP1 | target | Cinobufagin | MMP12 | target | Resibufogenin | FGFR1 | target |
| COVID-19 | OAT | disease | Bufalin | NR1I2 | target | Cinobufagin | ZAP70 | target | Resibufogenin | JAK2 | target |
| COVID-19 | KIF11 | disease | Bufalin | ERBB4 | target | Cinobufagin | AKT2 | target | Resibufogenin | MMP2 | target |
| COVID-19 | SETD7 | disease | Bufalin | CMA1 | target | Cinobufagin | MMP2 | target | Resibufogenin | MMP12 | target |
| COVID-19 | BST1 | disease | Bufalin | RARB | target | Cinobufagin | RARB | target | Resibufogenin | RARA | target |
| COVID-19 | PDE3B | disease | Bufalin | MMP2 | target | Cinobufagin | DCK | target | Resibufogenin | PDE5A | target |
| COVID-19 | MMP8 | disease | Bufalin | IL2 | target | Cinobufagin | VDR | target | Resibufogenin | VDR | target |
| COVID-19 | GSTT2 | disease | Bufalin | ZAP70 | target | Cinobufagin | HCK | target | Resibufogenin | AKT2 | target |
| COVID-19 | BACE1 | disease | Bufalin | TGM3 | target | Cinobufagin | NR1I2 | target | Resibufogenin | TGM3 | target |
| COVID-19 | PNMT | disease | Bufalin | RARA | target | Cinobufagin | RXRB | target | Resibufogenin | PADI4 | target |
| COVID-19 | PADI4 | disease | Bufalin | JAK2 | target | Cinobufagin | DPEP1 | target | Resibufogenin | NR1I2 | target |
| COVID-19 | EPHB4 | disease | Bufalin | HCK | target | Cinobufagin | TGM3 | target | Resibufogenin | MMP3 | target |
| COVID-19 | MMP3 | disease | Bufalin | AKT2 | target | Cinobufagin | FABP4 | target | Resibufogenin | FABP4 | target |
| COVID-19 | FABP6 | disease | Bufalin | VDR | target | Cinobufagin | FABP5 | target | Resibufogenin | RXRB | target |
| COVID-19 | HINT1 | disease | Bufalin | SETD7 | target | Cinobufagin | IL2 | target | Resibufogenin | IL2 | target |
| COVID-19 | ZAP70 | disease | Bufalin | RXRB | target | Cinobufagin | BST1 | target | Resibufogenin | BST1 | target |
| COVID-19 | ERBB4 | disease | Bufalin | GRB2 | target | Cinobufagin | ACADM | target | Resibufogenin | CMA1 | target |
| COVID-19 | HCK | disease | Bufalin | BST1 | target | Cinobufagin | KIT | target | Resibufogenin | GRB2 | target |
| COVID-19 | PDPK1 | disease | Bufalin | ACADM | target | Cinobufagin | RARA | target | Resibufogenin | DCK | target |
| COVID-19 | RARG | disease | Bufalin | PADI4 | target | Cinobufagin | GRB2 | target | Resibufogenin | KIT | target |
| COVID-19 | FABP7 | disease | Bufalin | TGFB2 | target | Cinobufagin | SETD7 | target | Resibufogenin | SETD7 | target |
| COVID-19 | PDE4D | disease | Bufalin | STAT1 | target | Cinobufagin | TGFB2 | target | Resibufogenin | HINT1 | target |
| COVID-19 | APOA2 | disease | Bufalin | KIT | target | Cinobufagin | GSTT2 | target | Resibufogenin | GSTT2 | target |
| COVID-19 | PDE5A | disease | Bufalin | GSTM1 | target | Cinobufagin | STAT1 | target | Resibufogenin | TGFB2 | target |
| COVID-19 | DAPK1 | disease | Bufalin | GSTM2 | target | Cinobufagin | CASP1 | target | Resibufogenin | CASP1 | target |
| COVID-19 | PDE4B | disease | Bufalin | OAT | target | Cinobufagin | OAT | target | Resibufogenin | OAT | target |
| COVID-19 | CMA1 | disease | Bufalin | HINT1 | target | Cinobufagin | HINT1 | target | Resibufogenin | GSTM1 | target |
| COVID-19 | TGM3 | disease | Bufalin | CASP1 | target | Cinobufagin | GSTM2 | target | Resibufogenin | STAT1 | target |
| COVID-19 | RXRB | disease | Bufalin | DAPK1 | target | Cinobufagin | GSTM1 | target | Resibufogenin | GSTM2 | target |
| COVID-19 | DPEP1 | disease | Bufalin | GSTT2 | target | Cinobufagin | DAPK1 | target | Resibufogenin | DAPK1 | target |
